# Supplementary material for: Tea intake or consumption and the risk of dementia: a meta-analysis of prospective cohort studies
Source: PeerJ. 2023 Jul 18;11:e15688. doi: 10.7717/peerj.15688 (PMC10361076; doi:10.7717/peerj.15688)
Supplement: Supplemental Information 3 [file peerj-11-15688-s003.docx]

**1The rationale for conducting the systematic review / meta-analysis;**

Dementia is a group of neurodegenerative disorders characterized by persistent deterioration of cognitive function, including mainly Alzheimer's disease and vascular dementia. There is no doubt that dementia has become one of the priorities of health services worldwide. The latest data showing that An estimated 6.7 million Americans age 65 and older are living with Alzheimer's dementia today. This number could grow to 13. 8 million by 2060 barring the development of medical breakthroughs to prevent, slow or cure AD. What's worse, a Mendelian randomization study showed a close correlation between COVID-19 hospitalization and AD, which Consistent with another study that found that COVID-19 induced olfactory function decline was similar to AD and Lewy body dementia decline. These suggest that there may be a wave of post-COVID-19 dementia in the future.

There is no effective treatment drug for dementia, though the great advances in the diagnostic identification of dementia over the past few decades, Cholinesterase inhibitors donepezil, galantamine, and memantine drugs each have limitations and can only improve symptoms and delay the course of the disease.Other non-pharmacological interventions such as music therapy, pet therapy and gardening therapy are also mainly used to reduce the symptoms of dementia rather than to cure the disease.

The 2020 *Lancet* Commission identified several potentially modifiable risk factors to prevent and ameliorate dementia, including smoking, obesity, depression, physical inactivity, diabetes, low social contact, excessive alcohol consumption et al., a dozen factors. Controlled lifestyle and other factors which refers to reduce the incidence of dementia might be the global focus on dementia prevention and dementia management. Moreover,tea as the second best-selling drink in daily life, has many effects such as removing free radicals, fighting oxidation, promoting metabolism and protecting nerves. Cohort studies and randomized controlled studies have reported the efficacy of guan tea in cardiovascular diseases, diabetes, obesity and other diseases.The previous meta researches also reviewed the relationship between tea and AD, but did not find the importance of tea in the prevention of dementia. Recent high-quality literature on the relationship between tea and AD met the basic conditions of meta-analysis, Thus, this study systematically reviewed the relevant literature of tea and all-cause dementia, AD, and VaD to provide more evidence for the preventive management of clinical dementia

1. **The contribution that it makes to knowledge in light of previously published related reports, including other meta-analyses and systematic reviews**

There are Little meta reports about the relationship of tea intake between all-cause dementia, AD and and VaD. And most doping other factors such as coffee did not fully verify the positive effect of tea in preventing dementia, Kakutani et al include three cohorts and five cross-sectional studies including while only two cohort studies and four cross-sectional studies support the positive effect of green tea,in contrast，our study based on the principle of meta-analysis from the quantitative perspective of tea intake and all-cause dementia, AD, and VaD. In the systematic evaluation by Panza et al, several cross-sectional and longitudinal population-based studies showed a protective effect of coffee, tea and caffeine use against cognitive impairment / decline in later life, however such a positive effect was not found in the cognitive domain. In ran et aldose-response meta-analysis, Green tea consumption was a significant protective factor for cognitive health, with one cup of tea per day brings a 6% reduction in risk of cognitive deficits,we have similar conclusion. Our meta-analysis based on prospective, long-term follow-up cohort studies suggests that tea (green and black) intake or consumption has a potential role in reducing the risk of all-cause dementia, AD and VD. More interestingly,tea intake or consumption may reduce the risk of all-cause dementia in middle-aged and older adults, suggesting that tea intake and consumption may be a modulable lifestyle strategy to prevent the onset of dementia.In conclusion, our study reveals that tea has significant potential as an active lifestyle for dementia prevention in future dementia prevention management.
