# Supplementary material for: Tea intake or consumption and the risk of dementia: a meta-analysis of prospective cohort studies
Source: PeerJ. 2023 Jul 18;11:e15688. doi: 10.7717/peerj.15688 (PMC10361076; doi:10.7717/peerj.15688)
Supplement: Supplemental Information 4 [file peerj-11-15688-s004.doc]

**Supplementary**

**Table 1~3: Details of the Literature Search Strategy**

(1) PubMed (November 1, 2022)

| Search | Query | Results |
| --- | --- | --- |
| 1 | (("Dementia"[Mesh]) OR "Alzheimer Disease"[Mesh]) OR "Cognition Disorders"[Mesh] | 271,053 |
| 2 | (((((((Dementia*[Title/Abstract]) OR (Cognition Disorder*[Title/Abstract])) OR (Alzheimer Disease*[Title/Abstract])) OR (cognitive disorder*[Title/Abstract])) OR (cognition impairment*[Title/Abstract])) OR (cognitive dysfunction*[Title/Abstract])) OR (Alzheimer Dementia*[Title/Abstract])) OR (Amentia*[Title/Abstract]) | 170,135 |
| 3 | ((("Dementia"[Mesh]) OR "Alzheimer Disease"[Mesh]) OR "Cognition Disorders"[Mesh]) OR ((((((((Dementia*[Title/Abstract]) OR (Cognition Disorder*[Title/Abstract])) OR (Alzheimer Disease*[Title/Abstract])) OR (cognitive disorder*[Title/Abstract])) OR (cognition impairment*[Title/Abstract])) OR (cognitive dysfunction*[Title/Abstract])) OR (Alzheimer Dementia*[Title/Abstract])) OR (Amentia*[Title/Abstract])) | 330,873 |
| 4 | "Tea"[Mesh] | 12,296 |
| 5 | (tea [Title/Abstract]) OR ("Tea"[Mesh]) | 37,912 |
| 6 | (((("Dementia"[Mesh]) OR "Alzheimer Disease"[Mesh]) OR "Cognition Disorders"[Mesh]) OR ((((((((Dementia*[Title/Abstract]) OR (Cognition Disorder*[Title/Abstract])) OR (Alzheimer Disease*[Title/Abstract])) OR (cognitive disorder*[Title/Abstract])) OR (cognition impairment*[Title/Abstract])) OR (cognitive dysfunction*[Title/Abstract])) OR (Alzheimer Dementia*[Title/Abstract])) OR (Amentia*[Title/Abstract]))) AND ((tea[Title/Abstract]) OR ("Tea"[Mesh])) | 440 |

(2) Cochrane Library (November 1, 2022)

| **Search** | **Query** | **Results** |
| --- | --- | --- |
| #1 | MeSH descriptor: [Alzheimer Disease] explode all trees | 3,838 |
| #2 | MeSH descriptor: [Dementia] explode all trees | 6,831 |
| #3 | MeSH descriptor: [Cognition Disorders] explode all trees | 6,167 |
| #4 | #1 OR #2 OR #3 | 11,688 |
| #5 | (Cognition Disorder*):ti,ab,kw OR (Dementia*):ti,ab,kw OR (Alzheimer Disease*):ti,ab,kw OR (cognitive disorder*):ti,ab,kw OR (cognition impairment*):ti,ab,kw | 60,783 |
| #6 | (cognitive dysfunction*):ti,ab,kw OR (Alzheimer Dementia*):ti,ab,kw OR (Amentia*):ti,ab,kw | 13,268 |
| #7 | #4 OR #5 OR #6 | 63,634 |
| #8 | MeSH descriptor: [Tea] explode all trees | 563 |
| #9 | (tea):ti,ab,kw | 4,286 |
| #10 | #8 OR #9 | 4,286 |
| #11 | #7 AND #10 | 146 |

(3) Embase (November 1, 2022)

| **Search** | **Query** | **Items found** |
| --- | --- | --- |
| #1 | 'dementia'/exp OR 'alzheimer disease'/exp OR 'cognitive defect'/exp | 580,950 |
| #2 | 'cognition disorder*':ab,ti OR 'dementia*':ab,ti OR 'alzheimer disease*':ab,ti OR 'cognitive disorder*':ab,ti OR 'cognition impairment*':ab,ti OR 'cognitive dysfunction*':ab,ti OR 'alzheimer dementia*':ab,ti OR 'amentia*':ab,ti | 234,834 |
| #3 | #1 OR #2 | 610,959 |
| #4 | 'tea'/exp | 22,932 |
| #5 | 'tea':ab,ti | 45,000 |
| #6 | #4 OR #5 | 49,358 |
| #7 | #3 AND #6 | 959 |
